# How to manage children with the eye signs of vitamin A deficiency

**Published:** 2013

**Authors:** Clare Gilbert

**Affiliations:** Co-director: International Centre for Eye Health, Disability Group, London School of Hygiene and Tropical Medicine, London, UK.

**Figure:**
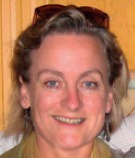
Clare Gilbert

The principles of managing children with the eye signs of vitamin A deficiency (VAD) – xerophthalmia – are:

1. correct the deficiency
2. manage the eye manifestations.

The initial focus should always be on correcting the VAD as children with any signs of xerophthalmia are at an increased risk of dying.

## Correcting the deficiency

Children with any of the features of xerophthalmia should be assumed to be markedly vitamin A deficient, with low stores of retinol in their livers. They need multiple doses of vitamin A to restore their serum levels and to boost their liver stores.

The recommended treatment for a child with any of the eye signs of VAD is shown in Table 1. Explain to the child's mother that three doses are needed. Watch the child being given the first dose to make sure he/she takes it, and give the mother the other doses for the next day and two weeks later. If the child is very sick and cannot swallow, intramuscular preparations are available. In cases of acute deficiency with ocular manifestations, parenteral/intramuscular injection of 50,000 international units of **water soluble** vitamin A is very useful, giving immediate improvement both in the ocular and systemic conditions.

## Managing the eye manifestations

### Corneal ulceration

Disentangling different causes of corneal ulcers in children (Table 2) can be challenging, as they can be due to a variety of causes, such as bacterial or fungal infections, herpes simplex infection, the use of harmful traditional eye remedies or some forms of trauma (e.g. burns from acid or hot fluids) as well as VAD. In the case of ulcers from VAD the diagnosis can be more difficult. There may be secondary infection, which makes the eyes red and painful with discharge, and the mother may have used harmful traditional eye remedies which can alter the appearance of the ulcer.

**Table:** Table 1. Recommended treatment of children with any of the eye signs of vitamin A deficiency

**Age of the child**	**Dose of vitamin A (IU)**	**Frequency**
<6 months	50,000	Day 1, day 2 and day 14
6-12 months	100,000	Day 1, day 2 and day 14
>12 months	200,000	Day 1, day 2 and day 14
IU = International units of retinyl palmitate

Take a careful history and examine the eyes carefully. **If in doubt, give high-dose vitamin A as well as other treatment**.

### Corneal scarring

If a child has bilateral, dense central corneal scars, an optical iridectomy (surgical removal of a small segment of peripheral iris through a small incision at the limbus) can dramatically improve the visual function (Figure 1).

This is usually only indicated, however, if both eyes are blind and there is a wide enough area of clear peripheral cornea. The operation is quick and easy and usually has no complications.

**Figure:**
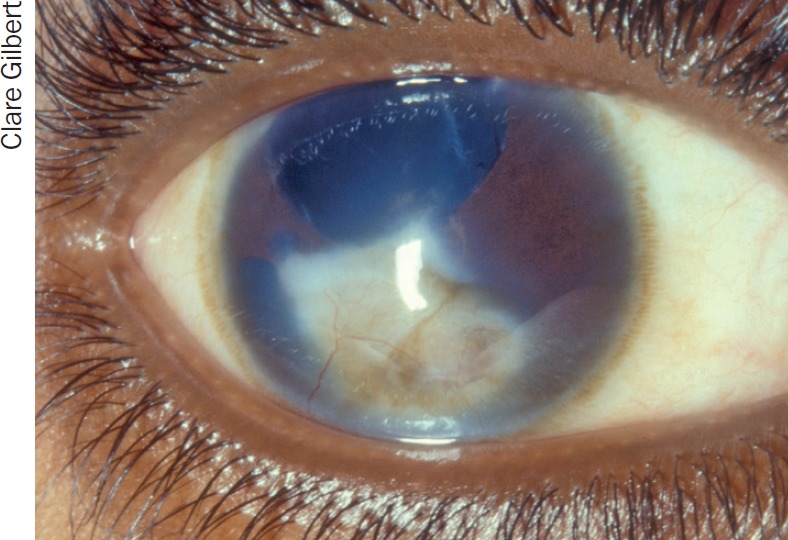
Figure 1. Optical iridectomy for central corneal scarring

One of the main challenges of corneal grafting in children with corneal scarring is that the chance of rejection is extremely high.^1^ The scars are vascularised and the cornea will have lost its ‘immune privilege’. Corneal grafting in these children also requires healthy young donor corneas, highly experienced corneal surgeons, parents who understand and will be compliant with instilling eye drops after surgery (possibly for months), and who will bring their child back for regular follow-up for months after surgery, as well as ophthalmologists who understand visual development in children.

As most children with corneal scars will be amblyopic (because the visual system did not develop normally), parents need to understand that visual acuity may be poor after a graft or iridectomy; however, the child's overall visual function may improve. Children in whom surgery is not possible should be referred for rehabilitation.

### Staphyloma and phthisis bulbi

Staphyloma (forward bulging of a badly damaged cornea) is often very uncomfortable or painful. If the child has a painful staphyloma the eye should be removed. Phthisical eyes (eyes that have shriveled up after severe eye disease) can be very disfiguring, and removing the eye and replacing it with an artificial eye can improve the appearance.

**Table:** Table 2. Differentiating between vitamin A deficiency and other causes of corneal ulceration

**Likely cause**	**From the history**	**From examination**
Vitamin A deficiency more likely if…	Recent illness such as measles, diarrhoea or other illness associated with fever No medication of any kind has been put in the eye(s) No history of eye injury Child not adequately breastfed The mother is poor and malnourished herself	Bilateral ulceration The ulcer has a punched-out appearance, or may even be full thickness with protrusion of the iris
Other causes more likely if …	History of trauma or hot fluid or acid getting into the eyes	Only one eye is affected and the other eye is entirely normal
